# Skin Carotenoid Status and Diet Quality in Professional Rugby League Players: An Exploratory Longitudinal Study

**DOI:** 10.3390/nu18142219

**Published:** 2026-07-08

**Authors:** Jessame Stepto, Alana Francis, Karen Charlton, Bridget Kelly, Joel C. Craddock

**Affiliations:** 1 School of Medical, Indigenous and Health Sciences, Faculty of Science, Medicine and Health, University of Wollongong, Wollongong, NSW 2500, Australia; 2 Manly Warringah Sea Eagles, 25 Federal Parade, Brookvale, NSW 2100, Australia; 3 School of Health Sciences, University of Newcastle, Callaghan, NSW 2308, Australia; 4 Nutrition and Metabolic Health Research Program, Hunter Medical Research Institute, Newcastle, NSW 2305, Australia

**Keywords:** sports nutrition, diet quality, carotenoid, HEIFA-2013, rugby league

## Abstract

Background/Objectives: Diet quality is relevant to athlete health and recovery, yet practical methods for assessing dietary patterns in elite sporting environments remain limited. This exploratory study aimed to examine the relationship between skin carotenoid status and diet quality in male professional National Rugby League players. Methods: Skin carotenoids were assessed at three time points throughout the NRL season: pre-, mid-, and end-season. The Veggie Meter^®^ was used to assess carotenoids alongside a single 24 h dietary recall at each time point. Dietary data for each player were scored using the Healthy Eating Index for Australian Adults 2013 (HEIFA-2013) to calculate a score out of 100. Correlations were explored via Pearson correlation coefficients, and linear mixed models were used to compare data over the season. Results: Correlations between carotenoid status and diet quality score varied across time points: pre-season, r = 0.297, 95% CI −0.094 to 0.608, *p* = 0.196; mid-season, r = 0.451, 95% CI 0.108 to 0.698, *p* = 0.012; and end-season, r = 0.335, 95% CI −0.036 to 0.625, *p* = 0.076. Mean carotenoid scores did not differ significantly across the season: 294 in pre-season (*n* = 27), 314 in mid-season (*n* = 30), and 318 in end-season (*n* = 29), *p* = 0.16. Mean HEIFA-2013 scores also did not differ significantly across the season: 66.4 in pre-season, 64.6 in mid-season, and 62.6 in end-season, *p* = 0.38. Conclusions: This exploratory study provides preliminary evidence that skin carotenoid status may reflect aspects of diet quality in elite NRL players, although associations varied across time points and only reached statistical significance at mid-season. Non-invasive tools such as the Veggie Meter^®^ may complement traditional dietary assessment methods, although further validation is required in larger, adequately powered studies of elite athletes.

## 1. Introduction

Sports nutrition is a rapidly advancing discipline centered on optimizing athletic performance. Manipulation of macronutrients, including carbohydrates, fats, and proteins, alongside hydration and sport-specific supplements, is widely recognized as an essential element of performance nutrition [1]. Dietary practices are often monitored using methods such as diet histories and 24 h recalls [2], and can support muscle protein synthesis, reduce injury risk and improve physical performance [1]. Despite this strong performance focus, overall diet quality among athletes is often suboptimal, with many relying on convenient and highly processed foods to meet their high energy and nutrient demands [3].

Diet quality reflects both the diversity and overall nutritional adequacy of an individual’s dietary intake, encompassing contributions from macronutrients, as well as micronutrients, including minerals and antioxidants [4]. It is commonly assessed using validated diet quality indices (DQIs) [5,6]. Assessing diet quality is particularly relevant in athletic populations, where higher DQI scores have been linked to improved wellbeing and may support enhanced athletic performance [7]. Despite this, diet quality and micronutrient intake often receive less attention in sports nutrition even though they play important roles in supporting recovery, immune function and injury prevention [8,9].

Carotenoids, fat-soluble pigments found abundantly in fruits and vegetables, represent one such under-acknowledged group of micronutrients [10]. These compounds accumulate in the lipid bilayer of human skin cells [11] and are recognized for their anti-inflammatory and antioxidant properties helping to mitigate free radical damage and inflammatory processes associated with illness, physiological stress and poor diet [10,11]. Carotenoid concentrations correlate strongly with fruit and vegetable intake and can serve as a proxy for higher overall diet quality [12]. In the general population higher carotenoid intake has been linked to a reduced risk of chronic diseases such as cardiovascular disease and diabetes [13]. However, research specifically examining carotenoid benefits within athletic populations remains limited.

Among athletes, carotenoids may be relevant not only as markers of fruit and vegetable intake and broader diet quality [12], but also as compounds with antioxidant and anti-inflammatory properties [10,11] that may support immune function and recovery from exercise-related physiological stress [13,14]. This may be particularly important in elite sporting environments, where high training loads, repeated competition and travel can increase physiological stress and challenge immune function [14]. However, evidence specifically examining carotenoid status and its relationship with diet quality in elite athlete populations remains limited [15].

Traditionally, carotenoid status has been measured using blood serology [16], an approach that is invasive, time-consuming and costly. The Veggie Meter^®^, however, offers a non-invasive alternative using ultraviolet light-based spectroscopy to estimate skin carotenoid concentrations [17]. Validated across diverse populations, the device provides a rapid estimate of fruit and vegetable intake over the preceding six-week period in under 90 s [17]. Higher Veggie Meter^®^ scores (>400) have been associated with better diet quality and lower adiposity [18]. To our knowledge, to date only one study has simultaneously collected data on diet quality indices, carotenoids and body composition in athletes [15]; however, correlations between diet quality and carotenoid status were not examined.

In addition to dietary intake, other factors may influence skin carotenoid levels, including adiposity, smoking status, season of measurement, training load, and physiological stress. Lower skin carotenoid scores have been reported among individuals with higher adiposity, smokers, and during winter measurement periods [19,20], while high training loads and cumulative physiological stress may plausibly influence carotenoid status through increased oxidative turnover or altered inflammatory status [14,21]. Applications of the Veggie Meter^®^ in athletic populations remain scarce, underscoring the need for further investigation. This study aimed to examine the relationship between carotenoid status and diet quality in male professional National Rugby League (NRL) players. Specifically, diet quality was assessed using the Healthy Eating Index for Australian Adults 2013 (HEIFA-2013), skin carotenoid status was measured using the Veggie Meter^®^, and associations between diet quality and carotenoids were examined at three time points across the NRL season to explore seasonal patterns. 

## 2. Materials and Methods

**Figure 1:**
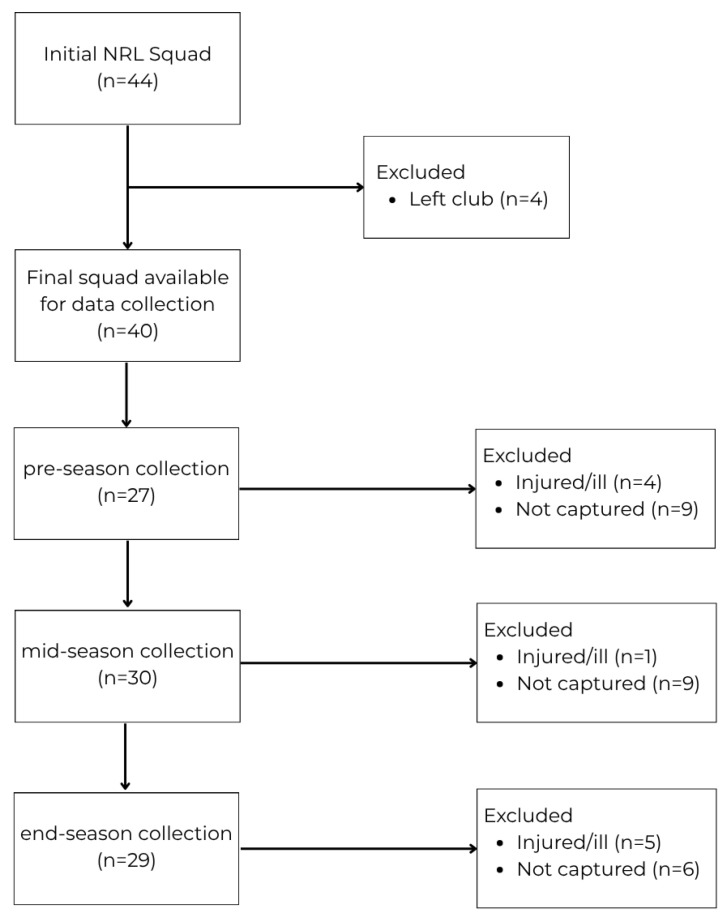
Flow of professional rugby league players included at pre-season, mid-season and end-season data collection time points.

This exploratory study was conducted over one NRL season in 2025, with three collection time points: pre (Jan), mid (May), and end of season (August). The available population was 40 male athletes from the Manly Sea Eagles first-grade squad. No a priori sample size calculation was undertaken, as this exploratory study used a convenience sample comprising the available first-grade playing squad. The sample therefore reflected the number of eligible players able to be assessed at each collection point within the operational constraints of the professional club environment. As player availability varied across collection points, time point comparisons were interpreted cautiously as exploratory seasonal differences. If a player reported illness or injury at any time point, their data for that corresponding collection time were removed from the analyses to reduce the potential influence of acute illness, injury, altered training load or physiological stress on dietary intake and carotenoid status (Figure 1). In the case of a player transferring clubs or departing the squad during the season, all data from that player were removed. Player characteristics, including gender, age, height and body weight at each collection, were also captured.

At each time point, measures of both carotenoid status and dietary intake were collected. The Veggie Meter^®^ was used to assess skin carotenoids, with three readings collected from each individual and an average score assigned at each time point. An accredited sports dietitian conducted a single face-to-face 24 h recall with each player at each time point. The 24 h recall captured all foods and beverages consumed during the preceding 24 h. All food data were entered into FoodWorks (version 10) and assessed using the AUSNUT 2011–2013 food composition database [22].

Each player’s dietary data were assessed using the HEIFA-2013, a validated diet quality index for Australian populations [23]. The HEIFA-2013 comprises 11 categories and generates a total score ranging from 0 to 100, assessing alignment with the Australian Dietary Guidelines (ADGs) [23]. The index includes positively scored categories (vegetables, fruits, grains, milk and milk alternatives, meat and meat alternatives, mono- and polyunsaturated fats, and water) and negatively scored categories (saturated fat, added sugars, sodium and alcohol). Positive categories are those where higher intake is encouraged, whereas negative categories are those in which higher intake is discouraged. For each category, scores are given depending on the player’s alignment with the recommended number of serves and serve sizes (Table 1).

To score a player’s diet quality using the HEIFA-2013 framework (Table 1), dietary data were converted into serves for each of the HEIFA-2013 categories. For categories that directly aligned with the ADG food groups (i.e., fruit and dairy), serves/100 g for each food item were obtained from the ADG serve sizes database [25]. Where a HEIFA-2013 category was unavailable (i.e., discretionary foods) or contained subcategories (i.e., vegetable sub-types), manual calculations were undertaken.

Because discretionary food serving sizes are defined by energy content (600 kJ per serve) rather than by weight in grams [26], items first needed to be standardized to a per 100 g serve basis before scoring. Discretionary foods were identified from the Australian Bureau of Statistics’ database via an 8-digit ID code [26]. The AUSNUT2011-2013 database was then used to obtain the kilojoule content per 100 g for each discretionary food item. As the Australian Dietary Guidelines define one discretionary serve as the amount of food containing 600 kJ [27], the kJ/100 g values were divided by 600 to derive the number of discretionary serves per 100 g for each item.

For HEIFA-2013 categories containing subcategories (e.g., vegetable sub-types), identification codes were used to group foods and calculate serves per 100 g. For example, foods with identification codes beginning with “202” in the ADG database were classified as orange vegetables. The weight of each food item reported by a player was multiplied by the serves-per-100 g value to calculate total serves per HEIFA-2013 category. Ready-to-drink fruit juices and juice concentrates were excluded from the fruit category in accordance with ADG recommendations [28] and were instead classified as discretionary foods. For HEIFA-2013 components not defined by ADG food groups (e.g., sodium, saturated fat and added sugars), nutrient intakes were calculated using the AUSNUT 2011-2013 database. Scores for each of the 11 categories were summed to provide a total diet quality score out of 100 (Table 1).

Statistical testing was completed using IBM SPSS (Version 30) [29]. Shapiro–Wilk tests were applied to determine normality [30]. Correlations between HEIFA-2013 and carotenoid scores were investigated using Pearson’s coefficient for parametric data. Spearman’s rank correlation was used for nonparametric data. To investigate differences in players’ carotenoid and diet quality scores across collection points, linear mixed models were used to account for repeated measures and missing data. However, because not all players contributed data at each time point, time point comparisons were interpreted as exploratory and may reflect both within-player variation and differences in participant composition across collection points.

## 3. Results

Participant flow across collection points is shown in Figure 1. Participant characteristics, including age, height, body weight and BMI at each collection point, are presented in Table 2. Among the included players, ages ranged from 19 to 36 years across the season, the mean height ranged from 186.5 to 187.6 cm, the mean body weight ranged from 99.4 kg to 103.6 kg, and the mean BMI ranged from 28.5 to 29.5 kg/m^2^ across collection points. Body weight and BMI differed significantly across collection points (Table 2). As player availability varied across collection points, observed differences may reflect both changes within individual players and differences in the players assessed at each time point.

Mean skin carotenoid scores were 294 in pre-season (*n* = 27), 314 at mid-season (*n* = 30), and 318 at end-season (*n* = 29) (Table 3). Differences between time points were not statistically significant (*p* = 0.16). Mean diet quality scores were 66.4 out of 100 in pre-season, 64.6 at mid-season, and 62.6 at end-season, with no statistically significant differences observed across the season (*p* = 0.38). HEIFA-2013 component scores varied across dietary domains. Categories including total vegetables, grains, meat and alternatives, dairy, water, mono- and polyunsaturated fats, discretionary foods, and added sugars generally scored closer to their maximum possible values, whereas categories including fruit intake and fruit variety, legumes, wholegrains, saturated fat, sodium, and selected vegetable subcategories, including orange vegetables, generally scored closer to their minimum possible values, indicating unfavorable intakes. Scores for green vegetables differed across time points, with higher scores observed at mid-season compared with pre-season (Table 4). Across the season, total energy and macronutrient intakes differed significantly, while changes in micronutrient intakes varied by nutrient. No statistically significant seasonal differences were observed for added sugars, saturated fat, fiber, or sodium (Table 3).

When analyzed at each time point, correlations between diet quality and carotenoid status varied across the season: pre-season, r = 0.297, 95% CI −0.094 to 0.608, *p* = 0.196; mid-season, r = 0.451, 95% CI 0.108 to 0.698, *p* = 0.012; and end-season, r = 0.335, 95% CI −0.036 to 0.625, *p* = 0.076. Only the mid-season association reached statistical significance.

## 4. Discussion

This study examined the relationship between skin carotenoid status and diet quality in male professional National Rugby League players, and is, to our knowledge, the first to apply the Veggie Meter^®^-derived carotenoid score alongside a validated diet quality index within an elite athletic population. While diet quality has previously been assessed in athlete cohorts, existing research has relied almost exclusively on self-reported dietary data and has rarely incorporated objective biomarkers of dietary exposure. Across three seasonal time points, neither diet quality nor skin carotenoid scores differed significantly. Correlations between diet quality and carotenoid status were modest and varied by time point, with only the mid-season association reaching statistical significance. These findings suggest that carotenoid status may provide some indication of overall diet quality; however, variation in the association across the season may also be influenced by unmeasured factors such as changes in body composition and training load, which were not assessed or controlled for in the present study.

Beyond examining the association between diet quality and carotenoid status, this study highlights the potential utility of objective, non-invasive carotenoid assessment within elite sporting environments. Diet quality is increasingly recognized as relevant to athlete health, recovery, and long-term wellbeing; however, routine assessment in professional sports remains challenging due to time constraints, respondent burden, and reliance on self-reported intake. The Veggie Meter^®^ offers a rapid, objective measure of carotenoid status that can be implemented alongside traditional dietary assessment. This assessment provides a practical indicator of carotenoid-rich dietary patterns without the need for blood sampling. In this context, skin carotenoid measurement may be a useful screening or monitoring tool, helping practitioners identify athletes with consistently lower micronutrient-dense food intakes and prioritize targeted dietary support. Importantly, the Veggie Meter^®^ does not replace comprehensive dietary assessment but rather complements diet quality indices by adding an objective biomarker that is feasible for repeated use across a competitive season. The successful integration of this technology within a professional NRL setting demonstrates its potential applicability as a complementary approach to nutrition assessment in high-performance sport.

Direct comparison with the existing literature is limited, as few studies have concurrently assessed diet quality and carotenoid status in athletic populations. Consistent with this, our recent scoping review of diet quality indices used in athletes identified a small and heterogeneous evidence base with most studies relying on dietary intake data and rarely incorporating objective biomarker validation [3]. Only one prior study has reported both outcomes in athletes; however, this work was conducted in collegiate swimmers, wrestlers, rowers, and gymnasts and did not quantify correlations between diet quality and carotenoid status due to limited dietary data [15]. In that cohort of 129 collegiate athletes, mean Healthy Eating Index-2015 scores averaged 71.0 out of 100, which is slightly higher than observed in our professional NRL cohort. Collegiate athletes scored well for fruit intake, whereas players in the current study scored poorly for both total fruit intake and fruit variety. While this may reflect genuine differences in dietary patterns between cohorts, underreporting is a recognized limitation of 24 h dietary recalls [31] and may partially explain these findings. Conversely, collegiate athletes scored poorly for unsaturated fats, whereas players in the present study achieved higher scores for mono- and polyunsaturated fats, reflecting regular consumption of foods such as fatty fish, vegetable oils, avocado, and nut-based products. Given the scarcity of athlete-specific studies quantifying diet quality-carotenoid relationships, broader evidence from general population cohorts provides important context for interpreting the observed association in the present study.

Evidence from general population studies indicates that overall diet quality, when operationalized using composite diet quality indices, is positively associated with carotenoid biomarkers. In a large adult cohort, Kim et al. (2019) demonstrated that higher diet quality, assessed using the Recommended Food Score, was significantly associated with higher total and individual plasma carotenoid concentrations in multivariable-adjusted models, and interpreted plasma carotenoids as objective indicators of overall dietary quality rather than a measure of the consumption of isolated food groups [32]. Additional studies have reported statistically significant associations between dietary pattern scores and serum carotenoids [32,33]. Furthermore, non-invasive assessment studies have shown that skin carotenoid status correlates strongly with plasma carotenoids, reinforcing the biological plausibility of using carotenoid status as an objective marker of carotenoid-rich dietary patterns in community samples [34].

Despite the limited comparative literature, additional insights can be drawn from both athlete studies and population-based cohorts. In a study of 9435 Australian adults aged 18 years and over, the average HEIFA-2013 score was reported to be 45.5 out of 100 [35]. Diet quality scores observed in the present cohort were consistently higher than population averages across all three collections, a finding that aligns with previous work indicating that athletes generally demonstrate higher diet quality than non-athletic populations [3]. Higher scores in discretionary foods, added sugars, meat and alternatives, water, dairy, and total grains contributed to this difference, reflecting dietary patterns commonly emphasized in applied sports nutrition practice [23]. High scores in these categories may reflect nutrition education that prioritizes adequate protein and carbohydrate intake, as well as hydration, to support training, recovery, and performance [1]. Consistent with this interpretation, an Australian study utilizing the Athlete Dietary Index reported substantially higher diet quality scores in elite athletes compared with age- and sex-matched population norms [36].

Despite generally higher overall diet quality, consistent patterns of suboptimal intake were observed across specific food groups, both in the present cohort and across the athlete literature. Intakes of protein, fat, and sodium are commonly reported as adequate or high in athletic populations, while wholegrains, fruit, and micronutrient-dense foods are frequently under-consumed [15,37,38]. These trends mirror findings synthesized in our scoping review [3], which identified recurring inadequacies in food groups central to dietary quality despite sufficient macronutrient intake. This may reflect the dominant focus of sports nutrition on macronutrient provision and energy availability, with comparatively less emphasis on food quality and micronutrient diversity. In applied elite sport settings, athletes may adjust intake to meet energy and performance demands by increasing meal frequency, portion sizes, carbohydrate-rich foods, protein foods, recovery snacks, sports drinks, gels and convenience-based options around training and competition [39,40]. While these strategies can support fueling and recovery, they may not necessarily improve broader dietary quality if increases in energy intake are achieved through lower-fiber, lower-micronutrient or more highly processed foods. This may partly explain why macronutrient-related targets appeared relatively well supported in the present cohort, while fruit variety, wholegrains, legumes, sodium and saturated fat remained less favorable [15].

While several findings aligned with previous research, two key aspects of the present results were unexpected. First, discretionary food and added sugar intake appeared relatively low in this cohort, contrasting with other Australian data on ultra-marathon runners that have reported poorer diet quality using the HEIFA-2013 [41], characterized by frequent discretionary food consumption. Another Australian exploratory study found that athletes reported consuming discretionary foods daily, most commonly citing taste and convenience as primary drivers of intake [42]. Given the reliance on single 24 h dietary recalls, social desirability bias and selective underreporting of foods perceived as unhealthy cannot be ruled out.

Second, diet quality and carotenoid status demonstrated minimal variation across the season, differing from previous studies of collegiate athletes that reported significantly lower carotenoid status and poorer diet quality during pre-season compared with in-season collections [15]. Seasonal declines in fruit and vegetable intake and overall diet quality during non-competitive periods have been attributed to greater dietary autonomy, reduced access to structured nutrition support, and environmental constraints [43,44,45]. Although fruit and vegetable intake and diet quality often improve during competitive periods due to increased dietetic support and performance-related motivation [37], such seasonal changes were not evident in the present cohort. This relative stability may reflect consistent access to nutrition support within the professional NRL environment; however, methodological factors, including sample size and limited dietary sampling, may also have reduced sensitivity to detect seasonal change.

Although the strength of associations varied by time point, a key contribution of this study was the successful implementation of a novel dietary assessment approach within a professional sporting environment. The Veggie Meter^®^ provided a rapid, non-invasive measure of carotenoid status that was feasible to integrate into routine testing, reducing participant burden and the logistical and financial constraints typically associated with blood-based biomarker assessment. This practicality addresses a common barrier to diet quality evaluation in elite sport and supports further investigation of skin carotenoid assessment as a feasible complementary measure in high-performance settings. The concurrent use of a validated and cost-effective dietary assessment method, the 24 h dietary recall, further enabled efficient data collection without reliance on participants’ literacy levels [46]. Diet quality was quantified using the HEIFA-2013, a validated index aligned directly with the Australian Dietary Guidelines, allowing comparison with comprehensive food- and nutrient-based reference data [23]. Unlike narrower indexing tools, the HEIFA-2013 captures broader aspects of dietary quality and guideline adherence, enhancing interpretability within applied sports nutrition contexts. Collectively, this approach facilitated engagement with players and high-performance staff around dietary quality, shifting emphasis beyond traditional performance metrics such as body composition towards a more holistic evaluation of athlete nutrition.

Several limitations should be acknowledged. No a priori sample size calculation was undertaken, and the relatively small sample size may have limited statistical power, increasing the possibility of Type II errors. In addition, adjusted analyses were not performed because relevant potential confounders, including body composition, adiposity, training load and physiological stress, were not measured or available at each time point. As a result, residual confounding cannot be excluded. Although height, body weight and BMI were reported, direct measures of body composition were not available; therefore, we were unable to determine whether differences in body weight or BMI reflected differences in fat mass, lean mass or hydration status. Dietary intake was assessed using a single 24 h dietary recall at each time point, a method known to be prone to recall bias and underreporting, with reported error rates of approximately 11–14% in general populations [31,47,48], with similar concerns regarding dietary misreporting in athletic populations. The Veggie Meter^®^ reflects carotenoid exposure over several weeks, whereas the 24 h recall reflects intake from a single day. This temporal mismatch may have attenuated observed associations between diet quality and carotenoid status. Social desirability bias may have further influenced reporting, particularly for foods perceived as unhealthy, potentially resulting in over- or underestimation of diet quality scores [49]. Operational constraints limited dietary assessment to a single recall per collection and required brief data collection windows of approximately 5–10 min per player, increasing susceptibility to day-specific bias, particularly given variation between training, rest, and competition days [50]. Consistent with prior work, weaker associations between skin carotenoids and diet quality have been reported when 24 h recalls are used as the primary dietary assessment tool, compared with more detailed methods such as food frequency questionnaires [51,52,53]. While FFQs may provide more stable estimates of habitual intake and stronger diet–biomarker relationships [18,54,55,56,57], their use was not feasible within the time constraints of a professional sporting environment. In addition, limitations specific to carotenoid assessment should be considered. Although players reporting recent illness were excluded, elevated physical and psychological stress may influence carotenoid status independently of dietary intake [17]. Furthermore, exclusion or non-capture of players due to illness, injury or availability may have introduced selection bias, as these individuals may have differed from the included players in dietary intake, training load, physiological stress or carotenoid status. High training loads, repeated competition, and cumulative stress across an NRL season may affect oxidative balance and immune function [21], potentially contributing to variability in carotenoid readings. As physical activity and training load were not directly assessed or controlled for at each time point, the extent to which these factors influenced the results cannot be determined within the present study.

Despite these limitations, this study provides a foundation for the integration of rapid, objective diet quality assessment within elite sport. Future research should aim to collect dietary data across multiple days, including the use of multiple-day food records where feasible, to better capture day-to-day variability and improve sensitivity to longitudinal changes in habitual intake across elite sporting seasons. Where feasible, the inclusion of more detailed, repeated or carotenoid-specific dietary assessment tools may strengthen observed associations between diet quality and carotenoid status [52,55]. Expanding research across different sporting codes, competitive levels, and female athlete populations could further enhance generalizability. Finally, future studies may explore whether longitudinal changes in diet quality and carotenoid status are associated with markers of performance, recovery, or health, thereby strengthening the applied relevance of objective dietary monitoring in high-performance sport.

## 5. Conclusions

Assessment of diet quality and carotenoid status remains under-represented within applied sports nutrition practice, despite its relevance to understanding athlete health, recovery and long-term wellbeing. This study demonstrates that objective skin carotenoid assessment can be implemented alongside a validated diet quality index in a professional sporting environment, supporting its feasibility as a practical, complementary approach to nutrition assessment in elite athletes. The use of a rapid, non-invasive tool such as the Veggie Meter^®^ may provide useful additional information about carotenoid-rich dietary patterns and help inform targeted nutrition support. However, these findings should be interpreted as preliminary, and further validation is required before skin carotenoid assessment can be considered a standalone tool for routine dietary monitoring in elite athletes. Future larger, adequately powered studies should incorporate repeated dietary assessments and adjustment for relevant confounding factors to better establish the utility of this approach in high-performance sport.

## Figures and Tables

**Table 1:** HEIFA-2013 scoring criteria used for adult male players *.

HEIFA Category	Criteria For Max. Score	Criteria for Min. Score	Criteria for Sub Scores
**Discretionary Food** where 1 serve = 600 kJ **	≤3 serves/d	≥3 serves/d	<3 serves/d = 10 3–3.9 = 7.5 4.0–4.9 = 5 5–5.9 = 2.5 ≥6 serves = 0
**Vegetables** Subcategories Green & Brassica Veg Orange Veg Starchy Veg Whole Veg Legumes where 1 serve = 75 g	>6 serves/d and variety consumed	0 serves/d, nil variety	≥6 serves/d = 5 4.8–<6 serves = 4 3.6–<4.8 serves = 3 2.4–<3.6 serves = 2 1.0–<2.4 serves = 1 No vegetables = 0 Vegetable variety/d: ≥1 serve green & brassica = 1 ≥1 serve orange = 1 ≥1 serve of starchy = 1 ≥1 serve of whole veg = 1 ≥0.5 serves of legumes = 1
**Fruit** where 1 serve = 150 g	≥2 serves/d and variety consumed	0 serves/d, nil variety	Total fruit serves/day: ≥2 serves = 5 ≥1.5–<2 serves = 3.75 ≥1–<1.5 serves = 2.5 ≥0.5–<1 serves = 1.25 ≥0–<0.5 serves = 0 Fruit variety score/d: 2 or more varieties = 5 points
**Grains & Cereals** Subcategories: Total grains & cereals Wholegrains where 1 serve = 500 kJ	≥6 serves/d and 50% wholegrains	≤6 and <50% wholegrains	Total cereals serves/d: 6 serves = 5 5 serves = 4.17 4 serves = 3.34 3 serves = 2.5 2 serves = 1.67 1 serve = 0.84 Wholegrain cereal serves/d: ≥3 serves = 5 ≥2.5–<3 serves = 4 ≥2–<2.5 serves = 3 ≥1.5–<2 serves = 2 ≥1–1.5 serve = 1 No wholegrain = 0
**Lean Meat and Alternatives** Includes meat, seafood, plant proteins and meat alternatives where 1 serve = 500–600 kJ	≥3 serves/d	0 serves/d	Meat/meat alternative serves/d: ≥3 serves = 10 ≥2.5 serves = 8 ≥2 serves = 6 ≥1.5 serves = 4 ≥1 serve = 2 ≤0.5 serves = 0
**Dairy and Alternatives** Includes dairy products and dairy alternatives where 1 serve = 500–600 kJ	≥2.5 serves/d	0 serves/d	Dairy/dairy alternatives/d: ≥2.5 serves = 10 ≥2 serves = 8 ≥1.5 serves = 6 ≥1 serve = 4 ≥0.5 serves = 2 No dairy/dairy alternative = 0
**Water**	50% of total beverage consumption from water	No water consumed	Proportion of water consumed relative to total beverages/d: ≥50% = 5 ≥40% = 4 ≥30% = 3 ≥20% = 2 ≥10% = 1 0% = 0 Note: category only scored out of 5
**Saturated Fats**	Total saturated fat intake ≤ 10% total energy intake	Total saturated fat intake ≥ 10% total energy intake	Saturated fat ≤10% of energy = 5 >10–12% of energy = 2.5 >12% total energy = 0
**Mono- and Polyunsaturated Fatty Acids (MUFA & PUFA)** where 1 serve = 250 kJ	4 serves/d	MUFA and PUFA none OR < 1 serve/d	PUFA and MUFA/d: 4 serves = 5 3–<4 serves = 3.75 2–<3 serves = 2.5 1–<2 serve =1.25 0–<1 serve = 0
**Sodium** Includes sodium in food items, used in cooking & at the table	≤920 mg/d	≥2300 mg/day	Sodium/d: 0 to 70 mmol (920–1610 mg) = 10 70 to 100 mmol (1610–2300 mg) = 5 ≥100 mmol (2300 mg) of Na/d = 0
**Added Sugars**	<15% Total energy intake/d	>20% Total energy intake/d	<15% total energy = 10 >15–<20% total energy = 5 >20% energy = 0
**Alcohol** where 1 serve = 1 standard drink	<2/d	>2 per day	≤2 per day = 5 >2 per day = 0 Note: category only scored out of 5

* Table 1 adapted and simplified from Roy et al. [23]. Only the recommended serves per day for adult males are shown, reflecting the study population. ** Serve sizes were defined according to the Australian Dietary Guidelines (ADGs) [24].

**Table 2:** Participant characteristics across seasonal collection time points.

	Pre-Season (*n* = 27)	Mid-Season (*n* = 30)	End-Season (*n* = 29)	*p*-Value
Age (years)	26.0 (24.2–27.8)	25.3 (23.7–26.9)	25.2 (23.4–27.0)	-
Height (cm)	187.6 (184.9–190.2)	186.9 (184.2–189.7)	186.5 (183.7–189.3)	-
Weight (kg)	103.6 (99.4–107.9)	99.8 (95.8–103.9) ^a^	99.4 (95.0–103.9)	0.002
BMI (kg/m^2^)	29.5 (28.3–30.7)	28.5 (27.6–29.5) ^a^	28.5 (27.5–29.5) ^b^	<0.001

Data are expressed as means with 95% confidence intervals. *p*-values represent the fixed effect of time from linear mixed models. ^a^ Statistically significant difference between pre-season and mid-season. ^b^ Statistically significant difference between pre-season and end-season.

**Table 3:** Skin carotenoid scores, diet quality scores, and nutrient intakes across seasonal collection time points.

	Pre-Season (*n* = 27)	Mid-Season (*n* = 30)	End-Season (*n* = 29)	*p*-Value
Carotenoid (score)	294.0 (269.67–318.4)	314.1 (290.9–337.2)	317.9 (292.6–343.1)	0.16
DQI (score/100)	66.4 (62.1–70.7)	64.6 (60.4–68.7)	62.6 (58.6–66.5)	0.38
Energy total (kJ)	13,050 (11,676–14,424)	10,303 (9330–11,276) *^a^*	10,183 (9080–11,287) *^b^*	<0.001
Carbohydrate (g)	242.9 (204.4–281.5)	191.5 (161.6–221.3) *^a^*	197.3 (167.2–227.4) *^b^*	0.01
Protein (g)	214.7 (187.9-241.5)	161.1 (143.8–178.5) *^a^*	164.7 (141.4–187.9) *^b^*	<0.001
Total fat (g)	139.0 (118.9–159.0)	111.9 (99.0–124.7) *^a^*	104.2 (89.8–118.6) *^b^*	<0.001
Saturated fat (g)	47.7 (40.5–54.9)	40.5 (34.5–46.4)	32.9 (26.5–44.1) *	0.05
Polyunsaturated fat (g)	18.2 (15.2–21.1)	12.4 (10.2–15.5) *^a^	15.4 (13.3–17.5)	0.02
Monounsaturated fat (g)	60.9 (51.6–70.2)	48.1 (42.2–54.0) *^a^*	43.5 (36.9–50.1) *^b^*	<0.001
Trans fat (g)	2.4 (1.5–3.0) *	2.4 (1.8–2.9)	1.8 (1.4–2.1)	0.15
Cholesterol (mg)	648 (487–1292) *	475 (368–875) *^a^	715 (559–870)	0.02
Added sugars (g)	15.7 (6.2–33.2) *	7.6 (0.0–25.9) *	14.4 (6.8–36.1) *	0.66
Dietary fiber (g)	23.0 (17.9–31.5) *	22.3 (18.6–26.0)	21.8 (18.4–25.1)	0.15
Thiamin (mg)	1.4 (1.0–2.1)	1.3 (1.0–2.0) *	1.4 (1.1–1.6)	0.18
Riboflavin (mg)	3.9 (3.3–4.5)	2.6 (2.2–3.1) *^a^*	2.5 (2.0–2.9) *^b^*	<0.001
Niacin (mg)	57.5 (48.4–66.5)	46.1 (39.7–52.5)	43.4 (36.5–50.3) *^b^*	0.03
Vitamin C (mg)	106.2 (52.1–191.4) *	79.6 (40.7–130.4) *	79.3 (46.0–118.4) *	0.10
Vitamin E (mg)	21.3 (16.2–25.1) *	15.6 (11.7–24.0) *	17.3 (14.0–20.6)	0.04
Vitamin B12 (μg)	11.6 (9.7–13.5)	9.2 (7.9–10.6)	8.0 (6.6–9.3) *^b^*	<0.001
Folate (μg)	665.2 (544.7–785.6)	484.0 (396.8–571.6) *^a^*	485.5 (391.2–579.7) *^b^*	0.01
Beta Carotene (μg)	2058.2 (767.3–11,170.4) *	1214.0 (545.3–8453.9) *	1138.4 (536.0–2660.7) *	0.19
Sodium (mg)	3058.1 (2471.4–3644.7)	2454.9 (1907.5–3002.3)	2707.6 (2262.1–3153.1)	0.19
Potassium (mg)	5640.3 (4935.3–6345.3)	4325.2 (3732.6–4917.8) *^a^*	4209.3 (3648.7–4769.9) *^b^*	<0.001
Magnesium (mg)	587.7 (517.5–658.0)	447.2 (387.2–507.3) *^a^*	442.9 (380.3–505.5) *^b^*	<0.001
Calcium (mg)	1346.4 (1102.0–1590.8)	1859.3 (1424.4–3166.1) *^a^	948.6 (763.4–1133.8) *^b^*	<0.001
Iron (mg)	17.0 (14.4–19.5)	13.9 (9.7–17.8) *	13.9 (9.6–16.7) *	0.26
Zinc (mg)	21.4 (18.2–24.5)	18.7 (15.9–21.6)	16.1 (12.1–22.4) *	0.34
Selenium (μg)	180.3 (156.1–204.5)	131.0 (110.7–151.2) *^a^*	142.9 (118.1–167.6)	<0.001
Linoleic (g)	13.4 (11.5–18.5)	10.5 (8.2–12.6) *	12.6 (11.0–14.3)	0.05
Alpha-linolenic (ALA) (g)	1.7 (1.3–2.4) *	1.3 (1.0–1.8) *	1.8 (1.6–2.1)	0.06
Eicosapentaenoic (EPA) (g)	0.2 (0.0–0.3) *	0.0 (0.0–0.2) *	0.1 (0.0–0.2) *	0.22
Docosapentaenoic (DPA) (g)	0.2 (0.2–0.2)	0.1 (0.1–0.2) *	0.1 (0.0–0.2) *	0.31
Docosahexaenoic (DHA) (g)	0.1 (0.1–0.4) *	0.1 (0.0–0.1) *	0.1 (0.1–0.1) *	0.12

All data are expressed as means with 95% confidence intervals unless otherwise stated. * Nonparametric data expressed as median and 25th–75th percentiles. *^a^* Statistically significant difference between pre-season and mid-season (Bonferroni adjusted). *^b^* Statistically significant difference between pre-season and end-season (Bonferroni adjusted). *p*-values represent the fixed effect of time from linear mixed models.

**Table 4:** Diet quality component scores based on the Healthy Eating Index for Australian Adults 2013 (HEIFA 2013) at each time point.

	Pre-Season (*n* = 27)	Mid-Season (*n* = 30)	End-Season (*n* = 29)	*p*-Value
Discretionary (score/10)	10.0 (0.0–10.0)	10.0 (2.5–10.0)	10.0 (2.5–10.0)	0.99
Total vegetables (score/5)	4.0 (2.0–5.0)	4.0 (2.0–5.0)	4.0 (2.0–5.0)	0.62
Green and brassica vegetables (score/1)	0.0 (0.0–1.0)	1.0 (0.0–1.0) *^a^*	0.0 (0.0–1.0)	<0.001
Orange vegetables (score/1)	0.0 (0.0–1.0)	0.0 (0.0–0.0)	0.0 (0.0–0.0)	0.63
Starchy vegetables (score/1)	1.0 (0.0–1.0)	0.5 (0.0–1.0)	1.0 (0.0–1.0)	0.74
Whole vegetables (score/1)	1.0 (0.0–1.0)	1.0 (0.0–1.0)	1.0 (1.0–1.0)	<0.001
Legumes as vegetables (score/1)	0.0 (0.0–0.0)	0.0 (0.0–0.0)	0.0 (0.0–0.0)	0.79
Total fruit (score/5)	1.3 (0.0–5.0)	1.3 (0.0–3.8)	1.3 (0.0–3.1)	0.85
Fruit variety (score/5)	0.0 (0.0–5.0)	0.0 (0.0–5.0)	0.0 (0.0–5.0)	0.73
Total grains (score/5)	4.2 (3.3–5.0)	3.8 (1.7–5.0)	3.3 (2.5–5.0)	0.63
Wholegrains (score/5)	0.0 (0.0–3.0)	0.0 (0.0–2.3)	0.0 (0.0–2.0)	0.53
Meat and alternatives (score/10)	10.0 (10.0–10.0)	10.0 (10.0–10.0)	10.0 (9.0–10.0)	0.63
Dairy and alternatives (score/10)	10.0 (6.0–10.0)	6.0 (2.0–10.0) *^a^*	6.0 (2.0–10.0)	0.02
% Water of total beverages (score/5)	5.0 (5.0–5.0)	5.0 (5.0–5.0)	5.0 (5.0–5.0)	0.25
% Energy saturated fat (score/5)	0.0 (0.0–2.5)	0.0 (0.0–2.5)	0.0 (0.0–3.8)	0.46
MUFA/PUFA (score/5)	5.0 (2.5–5.0)	4.4 (2.5–5.0)	5.0 (3.8–5.0)	0.59
Sodium (score/10)	0.0 (0.0–5.0)	5.0 (0.0–10.0)	0.0 (0.0–5.0)	0.08
Added sugars (score/10)	0.0 (0.0–5.0)	10.0 (10.0–10.0) *^a^*	10.0 (10.0–10.0) *^b^*	<0.001
Alcohol (score/5)	5.0 (5.0–5.0)	5.0 (5.0–5.0)	5.0 (5.0–5.0)	0.96
Total score (score/100) *	66.4 (62.1–70.7)	64.6 (60.4–68.7)	62.6 (58.6–66.5)	0.38

Data are presented as median and 25th–75th percentiles unless otherwise indicated. * Parametric data are presented as estimated marginal means with 95% confidence intervals derived from linear mixed models. *^a^* Statistically significant difference between pre-season and mid-season (Bonferroni adjusted). *^b^* Statistically significant difference between pre-season and end-season (Bonferroni adjusted). *p*-values represent the fixed effect of time from linear mixed models.

## Data Availability

The data presented in this study are not publicly available due to confidentiality and privacy restrictions associated with proprietary data owned by the Manly Warringah Sea Eagles Football Club.

## Abbreviations

The following abbreviations are used in this manuscript.

ADG	Australian Dietary Guideline
ALA	Alpha-linolenic acid
AUSNUT	Australian Food, Supplement and Nutrient Database
DHA	Docosahexaenoic acid
DPA	Docosapentaenoic acid
DQI	Diet Quality Index
EPA	Eicosapentaenoic acid
FFQ	Food Frequency Questionnaire
HEIFA-2013	Healthy Eating Index for Australian Adults 2013
HREC	Human Research Ethics Committee
kJ	Kilojoules
LMM	Linear Mixed Model
MUFA	Monounsaturated Fatty Acid
NRL	National Rugby League
PUFA	Polyunsaturated Fatty Acid
SPSS	Statistical Package for the Social Sciences
24hR	24 h dietary recall
